# Bacterial Pathogens Associated with Hidradenitis Suppurativa, France

**DOI:** 10.3201/eid2012.140064

**Published:** 2014-12

**Authors:** Hélène Guet-Revillet, Hélène Coignard-Biehler, Jean-Philippe Jais, Gilles Quesne, Eric Frapy, Sylvain Poirée, Anne-Sophie Le Guern, Anne Le Flèche-Matéos, Alain Hovnanian, Paul-Henry Consigny, Olivier Lortholary, Xavier Nassif, Aude Nassif, Olivier Join-Lambert

**Affiliations:** Université Paris Descartes, Sorbonne Paris Cité, Paris, France (H. Guet-Revillet, J.-P. Jais, E. Frapy, A. Hovnanian, O. Lortholary, X. Nassif, O. Join-Lambert);; Assistance-Publique Hôpitaux de Paris, Paris (H. Guet-Revillet, H. Coignard-Biehler, J-P. Jais, G. Quesne, S. Poirée, O. Lortholary, X. Nassif, O. Join-Lambert);; Institut National de la Santé et de la Recherche U1151 eq11, Paris (H. Guet-Revillet, E. Frapy, X. Nassif, O. Join-Lambert); Institut National de la Santé et de la Recherche U872 eq22 (J-P. Jais);; Institut National de la Santé et de la Recherche U 781, Paris (A. Hovnanian);; Institut Pasteur, Paris (A. Le Flèche-Matéos, A-S. Le Guern, P.-H. Consigny, A. Nassif)

**Keywords:** Hidradenitis suppurativa, Staphylococcus lugdunensis, Actinomyces, Streptococcus milleri group, anaerobic bacteria, Prevotella, infectious skin diseases, microbiota, opportunistic infections, antibiotic, antibacterial, antimicrobial, acne inversa, Verneuil disease, France, skin and soft tissue infections, bacteria

## Abstract

Hidradenitis suppurativa (HS) is a skin disease characterized by recurrent nodules or abscesses and chronic suppurating lesions. In the absence of clear pathophysiology, HS is considered to be an inflammatory disease and has no satisfactory medical treatment. Recently, prolonged antimicrobial treatments were shown to improve or resolve HS lesions. We prospectively studied the microbiology of 102 HS lesions sampled from 82 patients using prolonged bacterial cultures and bacterial metagenomics on 6 samples. *Staphylococcus lugdunensis* was cultured as a unique or predominant isolate from 58% of HS nodules and abscesses, and a polymicrobial anaerobic microflora comprising strict anaerobes, milleri group streptococci, and actinomycetes was found in 24% of abscesses or nodules and in 87% of chronic suppurating lesions. These data show that bacteria known to cause soft tissue and skin infections are associated with HS lesions. Whether these pathogens are the cause of the lesions or are secondary infectious agents, these findings support targeted antimicrobial treatment of HS.

## Abstract 2

*Staphylococcus lugdunensis* and anaerobic actinomycetes are associated with this skin infection.

Hidradenitis suppurativa (HS), also known as acne inversa and Verneuil disease, is a chronic disease of the apocrine gland–bearing areas of the skin (*1*). The prevalence of HS is estimated to be as high as 1% to 2% in the general population, and the disease has a serious effect on quality of life, placing it among the most distressing conditions observed in dermatology (*2*). It is therefore of major public health concern.

HS usually begins after puberty; the clinical severity of the disease varies among patients. Most patients have a mild form of the disease, manifested as painful large and deep-seated nodules. These lesions can resolve spontaneously, persist as “silent” nodules, or lead to abscess formation. In contrast, patients with severe HS have chronic, painful, suppurating lesions that persist for years. Chronic lesions usually involve multiple areas connected by inflamed and suppurating sinus tracts surrounded by hypertrophic scars.

The pathophysiology of HS is mostly unknown and probably multifactorial, including genetic, infectious, hormonal, and immunologic factors (*3*). Approximately one third of HS patients have a familial history of HS. Familial HS is transmitted with a dominant autosomal inheritance pattern, and mutations in the gamma secretase genes have been associated with a subset of familial cases (*4*,*5*). The current hypothesis is that the HS primary event is a hyperkeratinization of the follicular infundibulum, followed by follicular occlusion, dilatation and rupture; the spread of bacterial and cellular remnants would trigger the local inflammatory response (*3*). Previous microbiological studies found a wide range of bacteria sporadically associated with HS lesions: *Staphylococcus aureus*, *Streptococcus agalactiae,* coagulase-negative staphylococci, milleri group streptococci, anaerobes, and corynebacteria (*6*–*8*). Because of these confusing microbiological observations and the rapid relapse of HS lesions after standard antimicrobial drug treatments, bacteria were considered to be contaminants of primarily inflammatory lesions.

Recently, the rifampin-clindamycin drug combination was reported to substantively improve HS lesions (*9*,*10*), and our research team showed that complete healing of HS lesions can be obtained by using the rifampin-moxifloxacin-metronidazole drug combination (*11*). These findings suggest that suppurative lesions associated with HS may be of infectious origin. In this study, we performed an extensive microbiological study of HS lesions and identified 2 main profiles of opportunistic bacterial pathogens associated with HS lesions. These pathogens are commonly isolated from skin and soft tissue infections and are known to be sensitive to antimicrobial drug treatments used to obtain improvement or remission of HS lesions.

## Materials and Methods

### Patients and Lesions

In the Centre Médical de l’Institut Pasteur, a referral center for HS patients in France, microbiological samples are routinely obtained before initiation of antimicrobial treatment of HS patients. In this study, we performed a microbiological analysis of all HS lesions sampled from patients who consulted for the first time in our center for active HS during June 2007–February 2011 (Table 1). We excluded patients who received systemic or topical antibiotic drugs during the month before sampling. The clinical severity of lesions was assessed and designated by the same physician using the clinical severity staging of Hurley (*12*). Briefly, according to Hurley, stage 1 lesions correspond to nodules or abscesses, single or multiple, without sinus tracts or hypertrophic scars. Stage 2 lesions are single or multiple but nonconfluent lesions with sinus tracts and formation of scarring. Stage 3 lesions correspond to diffuse or nearly diffuse involvement of multiple interconnected sinus tracts or abscesses across an entire area.

**Table 1:** Characteristics of patients with hidradenitis suppurativa, France, Jun 2007–February 2011

Characteristics	Value
No. patients	82
Sex ratio (no. M/F)	0.33 (27/55)
Age, median y, ± SD	34 ± 9.5
Age, median y, ± SD at onset of disease	29 ± 9.9
Duration of Hurley stage,* y (range)	5.8 (0.5–21)
No. lesions	102
Clinical severity of lesions*	
Hurley stage 1	38
Hurley stage 2	45
Hurley stage 3	19
Location of lesions, no. (%)	
Inguinal fold and perineal area	32 (31)
Buttocks and thigh	19 (19)
Gluteal fold	13 (13)
Axilla	28 (27)
Breast	6 (6)
Trunk and neck	4 (4)
Microbiological samples, no.	183
Lesional samples†	125
Aspirates	11
Biopsy material	49
Lesional swabs	65
Perilesional control swabs	58

*Hurley staging: Hurley, stage 1 lesions correspond to nodules or abscesses, single or multiple, without sinus tracts or hypertrophic scars. Stage 2 lesions are single or multiple but non-confluent lesions with sinus tracts and formation of scarring. Stage 3 lesions correspond to diffuse or nearly diffuse involvement of multiple interconnected sinus tracts or abscesses across the entire area. †Each lesion was sampled 1 or 2 times. Open lesions with purulent drainage were sampled by swabbing of the purulent drainage. On consent of the patient, closed, nonsuppurative lesions were sampled only by biopsy or needle aspiration.

### Lesion Samples

We collected 2 types of lesion samples: 1) transcutaneous samples (from punch biopsies, ultrasonography guided biopsies, and needle aspirations) performed under strict asepsis using 5% povidone-iodine solution, and 2) swab specimens of superficial purulent drainage collected by using the Portagerm system (bioMérieux, Marcy l’Etoile, France) without aseptic preparation. Transcutaneous samples were collected only from patients who gave informed consent. Such samples were obtained from all closed abscesses or nodules and were also recommended for suppurative lesions. For suppurative lesions, we also suggested collecting purulent drainage by swab and collecting an additional control specimen at a 5-cm distance from the lesion, considering that biopsy may fail to reach the infectious site. No transport medium was used for punch biopsy specimens and purulent drainage collected by puncture. Samples were sent to the laboratory within 1 hour after sampling.

### Bacterial Cultures and Identification Methods

To grow anaerobic bacteria, we homogenized biopsy samples using a sterile porcelain mortar in 0.5 mL of Schaedler broth (bioMérieux, Marcy l’Etoile, France). Purulent drainage and swab specimens were directly discharged in 0.5 mL of Schaedler broth; 50 μL of the suspension was seeded on agar plates, including an Uriselect4 agar plate (Bio-Rad, Marnes-la-Coquette, France), a colistin-nalidixic acid (CNA) blood agar plate, and a Columbia blood agar plate (bioMérieux). Uriselect4 and CNA agar plates were incubated at 37°C under 5% CO_2_ for the isolation of aerobic and microaerophilic bacteria. Columbia agar plates were incubated anaerobically for 2 weeks. Cultures were analyzed at days 2, 7, and 15 by the same physician throughout the study. Anaerobic cultures were considered positive when the abundance or diversity of the bacterial culture was increased under anaerobic conditions. Plates were streaked by using the 4-phase pattern for isolation of predominant colonies. When the number of bacterial colonies was <200, colonies were counted; when >200 colonies, we assigned a colony count of 500 or 1,000 to bacterial colonies that reached the third or fourth quarter of the plate, respectively.

A maximum of 10 colonies per sample was identified by matrix-assisted laser desorption–time-of-flight mass spectrometry by using the Andromas system (*13*). When no identification was obtained, the 16S ribosomal RNA gene was sequenced. Altogether, 417 bacterial isolates were identified from the 162 culture-positive samples. Bacterial species were grouped in 12 categories (Technical Appendix Figures 1–5).

### Bacterial Metagenomics

To investigate more precisely the anaerobic microflora and to decipher whether nonculturable species could be associated with HS lesions, we performed a metagenomic study on 6 consecutive samples including 1 Hurley stage 1 abscess and 5 chronic suppurating lesions. We used sterile dry swabs to collect samples that were immediately frozen at −80°C. We extracted DNA extraction using the MagNa Pure technology (Roche Pharma, Boulogne-Billancourt, France). We amplified each sample using the eubacterial universal 16S primer set 27F/338R described by Fierer et al. that targets the V1 and V2 hypervariable regions of the small subunit of the ribosomal RNA gene (*14*). We used Platinium PCR SuperMix (Invitrogen, Carlsbad, CA, USA) to elicit amplification reactions. PCR products were purified and concentrated by using the UltraClean PCR Clean-up Kit (MoBio, Carlsbad, CA, USA). Samples were sent to GATC Biotech AG (Konstanz, Germany) to be pyrosequenced by using 454 Life Sciences sequencing (454 Life Sciences, a Roche Company, Branford, Connecticut, USA).

An average of 4,407 quality sequences (2,564–7,008 sequences) were obtained from each sample. We analyzed data using the QIIME software (*15*). Similar sequences were clustered into operational taxonomic units by using Uclust (*16*), with a minimum identity of 0.97. We assigned taxonomy using the RDP Classifier (*17*).

### Biostatistics

Statistical analyses were performed with R software version 3.01 (http://www.r-project.org). Hierarchical ascending classifications were performed to characterize sample profiles according to their bacterial composition. We computed the Euclidean distances between samples or species according to the bacterial abundance and then built dendrograms using the Ward aggregation criterion (*18*). Clustering results were figured in a heatmap chart (pheatmap R package, http://cran.r-project.org/web/packages/pheatmap/index.html) where samples and species are reordered according to the dendrograms.

We used the Fisher exact test to compare categorical variables. All tests were 2-sided; p values <5% were considered to be significant. We performed statistical analyses in R software.

## Results

We analyzed the microbiological test results of 102 typical HS lesions excised from 82 patients, comprising 38 nodules and abscesses (Hurley stage 1 lesions) and 64 chronic suppurating lesions (45 Hurley stage 2 and 19 Hurley stage 3 lesions) (Table 1). Altogether, we collected 125 lesional samples, including 11 needle aspirations, 49 biopsies performed under strict asepsis, and 65 swab samples of open suppurating lesions (Table 1). Twenty-three open suppurating lesions were sampled both by biopsy and swabbing. We obtained 58 perilesional control samples by swabbing normal perilesional skin 5 cm away from the lesion, bringing the total number of samples to 183.

### Identification of Microbiological Profiles Associated with HS Lesions

Culture results of the 183 samples are presented in Technical Appendix Figure 1. Of the 125 lesional samples, 106 yielded a positive culture. To identify single or multiple bacterial species specifically associated with HS lesions, hierarchical clustering was performed on these samples (Technical Appendix Figure 2). This strategy identified 2 microbiological profiles (Technical Appendix Figures 3–5).

The profile A group identified *Staphylococcus lugdunensis* as a unique or predominant isolate (Technical Appendix Figure 3). This group of samples comprised 24 lesion samples: 22/38 lesions were at Hurley stage 1 and 2/45 were at Hurley stage 2.

The profile B group was characterized by a mixed anaerobic flora composed of strict anaerobes and/or anaerobic actinomycetes and/or streptococci of the milleri group (Technical Appendix Figure 4). Various other bacteria, such as *S. aureus*, coagulase negative staphylococci, corynebacteria, *Enterobacteriaceae*, *Propionibacterium* spp., and *Enterococcus* spp., were inconstantly and in smaller amounts associated with the mixed anaerobic flora, especially when lesions were sampled by swabbing. Profile B organisms were found in 77 samples corresponding to 65 lesions.

Five samples from lesions did not correspond to the 2 identified microbiological profiles (Technical Appendix Figure 5). These samples were 2 Hurley stage 1 samples yielding pure culture of *Propionibacterium acnes* and 2 samples yielding corynebacteria, *Enterobacteriaceae*, and coagulase negative staphylococci. A pure culture of *Streptococcus pyogenes* was recovered from the fifth sample. A review of the medical file of the patient revealed that he was admitted for an acute infectious syndrome that is unusual in HS patients and probably corresponded to an acute superinfection.

Considering the polymicrobial nature of profile B, including bacterial species known to be nonpathogenic skin commensals, we aimed at determining the relevance of these bacteria in the pathogenic process. To achieve this goal, we first analyzed the culture results of 45 purulent drainage on swabs for which a perilesional control sample was obtained (Technical Appendix Figure 6). *S. aureus*, coagulase negative staphylococci, micrococcaceae, corynebacteria, *Propionibacterium* spp., *Enterobacteriaceae*, and *Enterococcus* spp. were isolated from both purulent drainage and perilesional controls. However, these organisms were isolated less frequently and in lower quantity from purulent drainage than from controls. Conversely, strict anaerobes, actinomycetes, and streptococci of the milleri group were recovered from almost all purulent drainage samples (42/45) and rarely from controls (6/45).

We next compared the culture results of 23 open suppurating lesions that were sampled by biopsy, needle aspiration under strict asepsis, or swabbing, for each of which a control perilesional swab was obtained (Technical Appendix Figure 7). Anaerobes, actinomycetes, and streptococci of the milleri group were isolated from purulent drainage, biopsies, and aspirations, but not from perilesional swabs. By contrast, *S. aureus*, non-*lugdunensis* coagulase-negative staphylococci, corynebacteria, *Enterobacteriaceae*, and *Propionibacterium* spp. were commonly isolated from purulent drainage and swabs but very rarely from biopsies.

These data demonstrate that anaerobes, actinomycetes, and streptococci of the milleri group are specifically associated with HS lesions. Other species isolated from purulent drainage samples were part of the normal skin flora and were likely to be present as contaminants.

### Correlation of Culture Results with Disease Severity and Lesion Topography

Our next task was to analyze the association of microbiological profiles with clinical severity, topography of the lesions, and gender (Table 2). Profile A was almost exclusively associated with Hurley stage 1 lesions, whereas profile B was predominantly associated with Hurley stage 2 and stage 3 lesions. Additionally, profile A tended to be associated with lesions of the breasts and buttocks.

**Table 2:** Microbiological profiles of 102 hidradenitis suppurativa lesions according to patient sex, clinical severity, and topography, France

Characteristics	Profile A	Profile B	No profile	Negative culture	p value*
Sex					0.2
M, n = 35	5	24	3	3	
F, n = 67	19	41	1	6	
Clinical severity of lesions†					2 × 10^–11^
Hurley stage 1, n = 38	22	9	2	5	
Hurley stage 2, n = 45	2	40	0	3	
Hurley stage 3, n = 19	0	16	2	1	
Topography of lesions					0.01
Inguinal fold, n = 32	5	24	1	2	
Buttock and thigh, n = 19	8	6	1	4	
Gluteal fold, n = 13	2	10	0	1	
Trunk, n = 4	1	2	1	0	
Breast, n = 6	4	2	0	0	
Axilla, n = 28	4	21	1	2	

*By Fisher exact test comparing the repartitions of profiles A and B. †Hurley staging: Hurley, stage 1 lesions correspond to nodules or abscesses, single or multiple, without sinus tracts or hypertrophic scars. Stage 2 lesions are single or multiple but nonconfluent lesions with sinus tracts and formation of scarring. Stage 3 lesions correspond to diffuse or nearly diffuse involvement of multiple interconnected sinus tracts or abscesses across the entire area.

Twelve patients had 2 or more lesions that had positive culture results (Table 3). Microbiological profiles were the same in 2 lesions of the same patient in 8 cases and different in 4 cases, indicating that microbiological profiles are not specific to a given individual.

**Table 3:** Microbiological profiles of 12 patients who had multiple hidradenitis suppurativa lesions, France*

Case-patient no.	Sample no.	Sampling method	Lesion site	Hurley stage†	Profile‡
30	172	Biopsy	L axilla	1	B
30	173	Swabbing	R axilla	2	B
36	180	Swabbing	Abdomen	2	B
36	181	Swabbing	Axilla	2	B
37	46	Swabbing	Breast	1	A
37	71	Biopsy	Buttock	1	A
39	7	Swabbing	Thigh	1	A
39	8	Swabbing	Inguinal fold	1	A
41	2	Biopsy	Thigh	1	A
41	110	Biopsy, swabbing	Inguinal fold	2	B
58	155	Swabbing	Axilla	3	B
58	156	Swabbing	Inguinal fold	3	B
60	85	Biopsy	Buttock	2	B
60	87	Swabbing	Inguinal fold	2	B
61	134	Needle aspiration	Buttock	1	A
61	137	Biopsy, swabbing	Inguinal fold	1	B
65	14	Swabbing	Pubis	1	B
65	16	Biopsy	Scrotum	1	B
76	177	Swabbing	L axilla	1	B
76	178	Swabbing	R axilla	2	B
76	179	Needle aspiration	Breast	1	A
78	89	Swabbing	L axilla	2	B
78	90	Biopsy	R axilla	2	B
82	96	Needle aspiration	Axilla	1	A
82	97	Swabbing	Inguinal fold	2	B

*For 12 case-patients, >1 lesion was analyzed. For 8 case-patients, samples yielded microbiological characteristics within the same profile, but in samples from lesions in case-patients 41, 61, 76, and 82, differing microbiological profiles were found; R, right; L, left. †Hurley staging: Hurley, stage 1 lesions correspond to nodules or abscesses, single or multiple, without sinus tracts or hypertrophic scars. Stage 2 lesions are single or multiple but nonconfluent lesions with sinus tracts and formation of scarring. Stage 3 lesions correspond to diffuse or nearly diffuse involvement of multiple interconnected sinus tracts or abscesses across the entire area. ‡Profile A: *S. lugdunensis* as unique or predominant pathogen; profile B: anaerobic microflora.

### Composition of Polymicrobial Anaerobic Flora as Assessed by Culture

The predominant anaerobic flora (1–7 different bacterial colonies per lesion) was studied in 36/62 profile B lesions (Table 4). A total of 95 anaerobic isolates were identified, including gram-positive cocci (predominantly *Anaerococcus* spp*., Peptoniphilus* spp., and *Finegoldia* spp.) and gram-negative rods (predominantly *Prevotella* spp., *Porphyromonas* spp., *Bacteroides* spp., and *Fusobacterium* spp.).

**Table 4:** Identification of strictly anaerobic bacteria cultured from 36 hidradenitis suppurativa lesions that had predominant anaerobic microflora, France

Gram type, genus, species	No. isolates
Gram-positive cocci	51
* Anaerococcus*	17
* A. vaginalis*	11
* A. prevotii*	4
* A. octavius*	1
* A. lactolyticus*	1
* Peptoniphilus*	15
* P. asaccharolyticus*	8
* P. harei*	3
* P. gorbachii*	1
* P. ivorii*	1
* P. lacrymalis*	1
Unidentified *Peptoniphilus* sp*.*	1
Other genera	19
* Finegoldia magna*	11
* Peptostreptococcus anaerobius*	4
* Parvimonas micra*	2
* Pediococcus acidilactici*	1
* Facklamia hominis*	1
Gram-negative rods	44
* Prevotella*	13
* P. bivia*	6
* P. disiens*	1
* P. intermedia*	1
* P. melaninogenica*	1
* P. salivae*	1
Unidentified *Prevotella* sp.	3
* Porphyromonas*	10
* P. asaccharolytica*	7
* P. somerae*	3
* Bacteroides*	9
* B. coagulans*	3
* B. ureolyticus*	3
* B. thetaiotaomicron*	2
* B. fragilis*	1
Other genera	19
* Fusobacterium*	8
* F. nucleatum*	7
* F. gonidiaformans*	1
* Dialister*	3
* D. pneumosintes*	2
* D. micraerophilus*	1
* Jonquetella*	1
* J. anthropi*	1

Predominant *Actinomyces* species were *A. turicensis* (30% of isolates), *A. radingae* (23% of isolates), *A. neuii* (14% of isolates), and *Actinobaculum schaali* (21% of isolates). Less frequently recovered species were *A. massiliae* (3 isolates), *A. europaeus* (2 isolates), *A. funkei* (1 isolate), and *A. urogenitalis* (1 isolate). In 14 lesions, >1 *Actinomyces* species (2 to 4) was identified.

Among streptococci of the milleri group, *Streptococcus anginosus* and *Streptococcus constellatus*, were recovered from 18 and 12 lesions, respectively. Both species were identified in the same samples in 2 cases.

### Composition of the Anaerobic Flora as Assessed by Metagenomics

We investigated the microbial diversity of 6 HS lesions by high-throughput sequencing. These lesions comprised 5 chronic suppurating lesions sampled by swabbing and 1 *S. lugdunensis* abscess sampled by needle aspiration (Figure). High-throughput sequencing confirmed that *Staphylococcus* was the far predominant taxon (99%) within the Hurley stage 1 abscess. In chronic suppurating lesions, anaerobic species (*Prevotella, Porphyromonas*, *Anaerococcus*, and *Mobiluncus* spp.) were the predominant taxa. Three bacterial orders were present in all samples: Bacteroidales (mainly *Prevotella* and *Porphyromonas*); Clostridiales (mainly *Peptoniphilus*, *Anaerococcus*, *Parvimonas*, *Dialister,* and *Finegoldia*); and Actinomycetale*s* (*Actinomyces*, *Actinobaculum*, and *Mobiluncus*).

**Figure:**
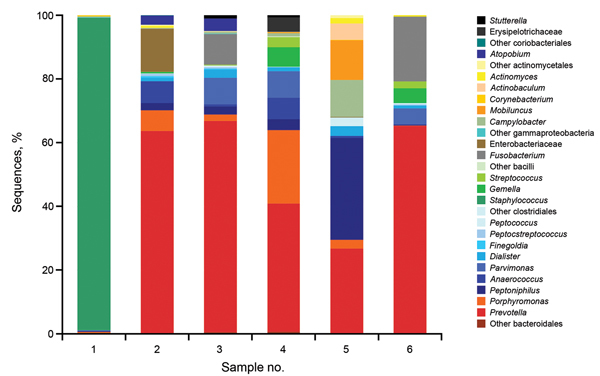
Microbial diversity of hidradenitis suppurativa (HS) lesions as assessed by high-throughput 454 sequencing. The bacterial diversity of 6 consecutive and representative HS lesions was assessed by high-throughput sequencing. Sample 1 corresponded to an acute *Staphylococcus lugdunensis* abscess sampled by needle aspiration. Samples 2–3 (swabs) corresponded to Hurley stage 2 lesions of the axilla and inguinal folds, respectively. Samples 4–6 (swabs) corresponded to Hurley stage 3 lesions of the inguinal, axilla and gluteal fold, respectively. *Staphylococcus* spp. represented >99% of sequences of sample 1, whereas *Prevotella* spp. represented the most abundant taxon in 4/5 of these chronic suppurative lesions.

Thus metagenomic data were consistent with culture results, facilitating a more exhaustive description of the anaerobic flora. It should be pointed out that metagenomics did not identify noncultivable bacteria associated with HS lesions.

Altogether, we identified 2 microbiological profiles specifically associated with HS lesions. Profile A was characterized by pure or predominant culture of *S. lugdunensi*s that was mostly associated with Hurley stage 1 lesions. Profile B was represented by a mixed flora composed of gram-negative and gram-positive strict anaerobes, anaerobic actinomycetes, and streptococci of the milleri group. This profile was mainly associated with open suppurating lesions observed in Hurley stages 2 and 3, but also with 24% of Hurley stage 1 lesions. No microbiological differences could be identified between Hurley stage 2 and 3 lesions by culture methods.

## Discussion

Identification of organisms and appropriate treatment are urgently needed to improve the quality of life of HS patients. An infectious process related to HS has been suspected for a long time (*19*). However, considering the polymicrobial nature of the cultures obtained from HS lesions and bacteria usually isolated from the skin microflora, it remained unclear whether bacterial factors were involved in the pathophysiology of HS.

We conducted a large prospective study of the microbiology of HS lesions among a cohort of 82 patients. Altogether, we studied 102 HS lesions using optimized sampling and culture methods. We used matrix-assisted laser desorption–time-of-flight mass spectrometry to identify the predominant microflora of lesions and of normal skin samples. The main advantage of this technique is that identification can be obtained to the species level within a few minutes for a very wide range of bacteria, including species of the normal microflora, anaerobes, and bacteria that are usually identified by molecular techniques.

This study demonstrates that 2 specific microbiological profiles, neither corresponding to the normal skin microflora, nor to usual skin pathogens (*S. aureus* and *S. pyogenes*) are associated with HS lesions. Two new bacterial pathogens species associated with HS lesions were identified: *S. lugdunensis* and anaerobic actinomycetes.

*S. lugdunensis* was mostly cultured from HS nodules and abscesses. *S. lugdunensis* is a skin commensal that primarily colonizes the lower extremities and inguino-perineal area, the latter including typical sites for HS lesions (*20*). *S. lugdunensis* seems to be an infrequent pathogen (*21*), but skin abscesses caused by this organism are similar to those caused by *S. aureus*, demonstrating a particular virulence compared with other coagulase negative staphylococci. This organism was initially described as a cause of post-surgical wound infections (*22*), suggesting that local predisposing factors are required for its pathogenicity.

Conversely, the majority of chronic suppurating lesions and a restricted number of mild HS lesions were associated with a predominant polymicrobial anaerobic microflora, including strict anaerobes, milleri group streptococci, and anaerobic actinomycetes. These bacteria are common inhabitants of the mouth and gastrointestinal tract (*23**–**25*). Strict anaerobes are usual colonizers but can cause secondary infections in patients who have local or systemic predispositions. They have been previously associated with HS lesions, and in various secondary skin infections including epidermal cysts (*26*–*28*). Strict anaerobes have been shown to grow synergistically and to cause severe infections especially when associated with other bacterial species, including milleri group streptococci, which were identified as a potential treatment target in HS in the 1980s (*19**,**29**–**31*), although they appeared to be unusual pathogens in other studies (*32*). Milleri group streptococci can be aggressive pathogens, leading to abscess formation at various sites of the body including the skin, thorax, and brain (*30*). They have been recently associated with chronic infectious conditions such as digestive fistula in patients who had vascular grafts or cystic fibrosis pulmonary infections (*33*,*34*).

Cultures of the vast majority of severe suppurating lesions produced anaerobic actinomycetes. Anaerobic actinomycetes are fastidious and aerotolerant species that grow slowly on rich media and provide pinpoint colonies after a 1-week culture period; they are also difficult to identify by using phenotypic methods. These factors probably show why they have not been cultured from HS lesions previously. Anaerobic actinomycetes have been associated with difficult-to-treat and relapsing skin and soft tissue infections. They can also cause severe infections such as endophthalmitis, bacteremia, and endocarditis (*35*–*38*). The closely related species *A. schaalii* is considered to be a uropathogen among persons ≥65 years of age and in patients with predisposing neurologic or local factors. *A. schaalii* has been recently associated with cellulitis and bacteremia (*39*).

Altogether, our study demonstrates that HS lesions are associated with bacterial species that can cause abscesses and severe infections. Compared with *S. aureus* or *S. pyogenes*, the low pathogenicity of these bacteria could account for the chronicity of suppuration of HS lesions that can last for years. In addition, the particular propensity of HS patients to develop recurrent or chronic skin infections highly suggests that HS is not primarily an infectious disease but a predisposing condition that allows these low pathogenic species to cause soft tissue and skin infections.

Histopathologic studies of noninflammatory areas of the skin of HS patients have shown the presence of dilated and distorted hair follicles. These anatomic abnormalities are thought to be caused by the hyperkeratinization of the follicular infundibulum, which can lead to dilatation and rupture and release of bacteria within the dermis (*40*). This event may predispose *S. lugdunensis* and anaerobic bacteria to cause nodules and abscesses in HS. Conversely, chronic suppurating HS lesions are deep abscesses drained by interconnected epithelialized sinus tracts. Colonization of these deep-seated lesions by anaerobic bacteria, streptococci of the Milleri group, and actinomycetes may account for chronic inflammation.

A limitation of our study is that HS patients who come to our center usually seek treatment for severe forms of the disease. Thus, the study population may not be representative of the HS patient population.

In summary, this study demonstrates that bacterial pathogens known to cause soft tissue and skin infections are found in HS lesions. The unexpected efficacy of wide-spectrum antimicrobial treatments for HS (*9*,*11*,*41*) highly suggests that these bacteria are partly causative agents for suppurative hidradenitis and should be considered to be treatment targets. These data open an avenue for future research on the pathophysiology of this disease, and provide a rational basis for clinical trials of treatment of HS.

Technical Appendix Microbiological results of 183 samples comprising 125 lesional samples and 58 control samples performed by swabbing normal perilesional skin. These results allowed the identification of 2 microbiological profiles associated with HS lesions.

## References

[1] Alikhan A, Lynch PJ, Eisen DB. Hidradenitis suppurativa: a comprehensive review. J Am Acad Dermatol. 2009;60:539–61, quiz 62–3. 10.1016/j.jaad.2008.11.911 19293006

[2] Wolkenstein P, Loundou A, Barrau K, Auquier P, Revuz J. Quality of life impairment in hidradenitis suppurativa: a study of 61 cases. J Am Acad Dermatol. 2007;56:621–3. 10.1016/j.jaad.2006.08.061 17097366

[3] Paus R, Kurzen H, Kurokawa I, Jemec GB, Emtestam L, Sellheyer K, What causes hidradenitis suppurativa? Exp Dermatol. 2008;17:455–6, discussion 7–72. 10.1111/j.1600-0625.2008.00712_1.x 18400064

[4] Pink AE, Simpson MA, Brice GW, Smith CH, Desai N, Mortimer PS, PSENEN and NCSTN mutations in familial hidradenitis suppurativa (acne inversa). J Invest Dermatol. 2011;131:1568–70. 10.1038/jid.2011.42 21412258

[5] Miskinyte S, Nassif A, Merabtene F, Ungeheuer MN, Join-Lambert O, Jais JP, Nicastrin mutations in French families with hidradenitis suppurativa. J Invest Dermatol. 2012;132:1728–30. 10.1038/jid.2012.23 22358060

[6] Highet AS, Warren RE, Weekes AJ. Bacteriology and antibiotic treatment of perineal suppurative hidradenitis. Arch Dermatol. 1988;124:1047–51. 10.1001/archderm.1988.01670070035015 3291777

[7] Lapins J, Jarstrand C, Emtestam L. Coagulase-negative staphylococci are the most common bacteria found in cultures from the deep portions of hidradenitis suppurativa lesions, as obtained by carbon dioxide laser surgery. Br J Dermatol. 1999;140:90–5. 10.1046/j.1365-2133.1999.02613.x 10215774

[8] Sartorius K, Killasli H, Oprica C, Sullivan A, Lapins J. Bacteriology of hidradenitis suppurativa exacerbations and deep tissue cultures obtained during carbon dioxide laser treatment. Br J Dermatol. 2012;166:879–83. 10.1111/j.1365-2133.2011.10747.x 22098253

[9] Gener G, Canoui-Poitrine F, Revuz JE, Faye O, Poli F, Gabison G, Combination therapy with clindamycin and rifampicin for hidradenitis suppurativa: a series of 116 consecutive patients. Dermatology. 2009;219:148–54 . 10.1159/000228334 19590173

[10] van der Zee HH, van der Woude CJ, Florencia EF, Prens EP. Hidradenitis suppurativa and inflammatory bowel disease: are they associated? Results of a pilot study. Br J Dermatol. 2010;162:195–7. 10.1111/j.1365-2133.2009.09430.x 19681876

[11] Join-Lambert O, Coignard H, Jais JP, Guet-Revillet H, Poiree S, Fraitag S, Efficacy of rifampin-moxifloxacin-metronidazole combination therapy in hidradenitis suppurativa. Dermatology. 2011;222:49–58 . 10.1159/000321716 21109728

[12] Hurley HJ. Axillary hyperhidrosis, apocrine bromhidrosis, hidradenitis suppurativa, and familial benign pemphigus: surgical approach. In: Roegnigk Randall K and Roegnigk Henry H, editors. Roegnigk & Roegnigk’s dermatologic surgery: principles and practice, second edition. Boca Raton, FL: CRC Press; 1996. p. 623–45.

[13] Bille E, Dauphin B, Leto J, Bougnoux ME, Beretti JL, Lotz A, MALDI-TOF MS Andromas strategy for the routine identification of bacteria, mycobacteria, yeasts, *Aspergillus* spp. and positive blood cultures. Clin Microbiol Infect. 2012;18:1117–25. 10.1111/j.1469-0691.2011.03688.x 22044600

[14] Fierer N, Hamady M, Lauber CL, Knight R. The influence of sex, handedness, and washing on the diversity of hand surface bacteria. Proc Natl Acad Sci U S A. 2008;105:17994–9. 10.1073/pnas.0807920105 19004758 PMC2584711

[15] Caporaso JG, Kuczynski J, Stombaugh J, Bittinger K, Bushman FD, Costello EK, QIIME allows analysis of high-throughput community sequencing data. Nat Methods. 2010;7:335–6 . 10.1038/nmeth.f.303 20383131 PMC3156573

[16] Edgar RC. Search and clustering orders of magnitude faster than BLAST. Bioinformatics. 2010;26:2460–1. 10.1093/bioinformatics/btq461 20709691

[17] Wang Q, Garrity GM, Tiedje JM, Cole JR. Naive Bayesian classifier for rapid assignment of rRNA sequences into the new bacterial taxonomy. Appl Environ Microbiol. 2007;73:5261–7 . 10.1128/AEM.00062-07 17586664 PMC1950982

[18] Ward JH. Hierarchical grouping to optimize an objective function. J Am Stat Assoc. 1963;58:236–44 http://www.jstor.org/stable/2282967. 10.1080/01621459.1963.10500845

[19] Highet AS, Warren RE, Staughton RC, Roberts SO. *Streptococcus milleri* causing treatable infection in perineal hidradenitis suppurativa. Br J Dermatol. 1980;103:375–82. 10.1111/j.1365-2133.1980.tb07259.x 7437303

[20] Bieber L, Kahlmeter G. *Staphylococcus lugdunensis* in several niches of the normal skin flora. Clin Microbiol Infect. 2010;16:385–8. 10.1111/j.1469-0691.2009.02813.x 19519842

[21] Frank KL, Del Pozo JL, Patel R. From clinical microbiology to infection pathogenesis: how daring to be different works for *Staphylococcus lugdunensis.* Clin Microbiol Rev. 2008;21:111–33. 10.1128/CMR.00036-07 18202439 PMC2223846

[22] Vandenesch F, Eykyn SJ, Etienne J, Lemozy J. Skin and post-surgical wound infections due to *Staphylococcus lugdunensis.* Clin Microbiol Infect. 1995;1:73–4 . 10.1111/j.1469-0691.1995.tb00449.x 11866733

[23] Murdoch DA. Gram-positive anaerobic cocci. Clin Microbiol Rev. 1998;11:81–120 . 9457430 10.1128/cmr.11.1.81 PMC121377

[24] Mejàre B, Edwardsson S. *Streptococcus milleri* (Guthof); an indigenous organism of the human oral cavity. Arch Oral Biol. 1975;20:757–62. 10.1016/0003-9969(75)90048-5 1061530

[25] Clarridge JE III, Zhang Q. Genotypic diversity of clinical *Actinomyces* species: phenotype, source, and disease correlation among genospecies. J Clin Microbiol. 2002;40:3442–8. 10.1128/JCM.40.9.3442-3448.2002 12202591 PMC130750

[26] Brook I. The role of anaerobic bacteria in cutaneous and soft tissue abscesses and infected cysts. Anaerobe. 2007;13:171–7. 10.1016/j.anaerobe.2007.08.004 17923425

[27] Brook I, Frazier EH. Aerobic and anaerobic microbiology of axillary hidradenitis suppurativa. J Med Microbiol. 1999;48:103–5 . 10.1099/00222615-48-1-103 9920133

[28] Brook I. Secondary bacterial infections complicating skin lesions. J Med Microbiol. 2002;51:808–12 . 12435058 10.1099/0022-1317-51-10-808

[29] Nagashima H, Takao A, Maeda N. Abscess forming ability of *Streptococcus milleri* group: synergistic effect with *Fusobacterium nucleatum.* Microbiol Immunol. 1999;43:207–16. 10.1111/j.1348-0421.1999.tb02395.x 10338189

[30] Belko J, Goldmann DA, Macone A, Zaidi AK. Clinically significant infections with organisms of the *Streptococcus milleri* group. Pediatr Infect Dis J. 2002;21:715–23. 10.1097/00006454-200208000-00002 12192158

[31] Hirai T, Kimura S, Mori N. Head and neck infections caused by *Streptococcus milleri* group: an analysis of 17 cases. Auris Nasus Larynx. 2005;32:55–8 . 10.1016/j.anl.2004.09.003 15882827

[32] Jemec GB, Faber M, Gutschik E, Wendelboe P. The bacteriology of hidradenitis suppurativa. Dermatology. 1996;193:203–6 . 10.1159/000246246 8944341

[33] Bonnet EP, Arista S, Archambaud M, Boot B, Clave D, Massip P, *Streptococcus milleri* group infection associated with digestive fistula in patients with vascular graft: report of seven cases and review. Infection. 2007;35:182–5 . 10.1007/s15010-007-6040-0 17565461

[34] Sibley CD, Sibley KA, Leong TA, Grinwis ME, Parkins MD, Rabin HR, The *Streptococcus milleri* population of a cystic fibrosis clinic reveals patient specificity and intraspecies diversity. J Clin Microbiol. 2010;48:2592–4. 10.1128/JCM.00414-10 20463160 PMC2897483

[35] Hall V. Actinomyces–gathering evidence of human colonization and infection. Anaerobe. 2008;14:1–7. 10.1016/j.anaerobe.2007.12.001 18222714

[36] Sabbe LJ, Van De Merwe D, Schouls L, Bergmans A, Vaneechoutte M, Vandamme P. Clinical spectrum of infections due to the newly described *Actinomyces* species *A. turicensis, A. radingae*, and *A. europaeus.* J Clin Microbiol. 1999;37:8–13 . 9854055 10.1128/jcm.37.1.8-13.1999 PMC84153

[37] Zautner AE, Schmitz S, Aepinus C, Schmialek A, Podbielski A. Subcutaneous fistulae in a patient with femoral hypoplasia due to *Actinomyces europaeus* and *Actinomyces turicensis.* Infection. 2009;37:289–91. 10.1007/s15010-008-7392-9 18854936

[38] von Graevenitz A. *Actinomyces neuii*: review of an unusual infectious agent. Infection. 2011;39:97–100. 10.1007/s15010-011-0088-6 21340579

[39] Beguelin C, Genne D, Varca A, Tritten ML, Siegrist HH, Jaton K, *Actinobaculum schaalii*: clinical observation of 20 cases. Clin Microbiol Infect. 2011;17:1027–31. 10.1111/j.1469-0691.2010.03370.x 20854424

[40] van der Zee HH, Laman JD, Boer J, Prens EP. Hidradenitis suppurativa: viewpoint on clinical phenotyping, pathogenesis and novel treatments. Exp Dermatol. 2012;21:735–9. 22882284 10.1111/j.1600-0625.2012.01552.x

[41] van der Zee HH, Boer J, Prens EP, Jemec GB. The effect of combined treatment with oral clindamycin and oral rifampicin in patients with hidradenitis suppurativa. Dermatology. 2009;219:143–7. 10.1159/000228337 19590174

