# Supplementary material for: Bacterial Pathogens Associated with Hidradenitis Suppurativa, France
Source: Emerg Infect Dis. 2014 Dec;20(12):1990–8. doi: 10.3201/eid2012.140064 (PMC4257786; doi:10.3201/eid2012.140064)
Supplement: Technical Appendix — Microbiological results of 183 samples comprising 125 lesional samples and 58 control samples performed by swabbing normal perilesional skin. These results allowed the identification of 2 microbiological profiles associated with HS lesions. [file 14-0064-Techapp-s1.pdf]

DOI: http://dx.doi.org/10.3201/eid2012.140064

Bacterial Pathogens Associated with Hidradenitis Suppurativa, France

Technical Appendix

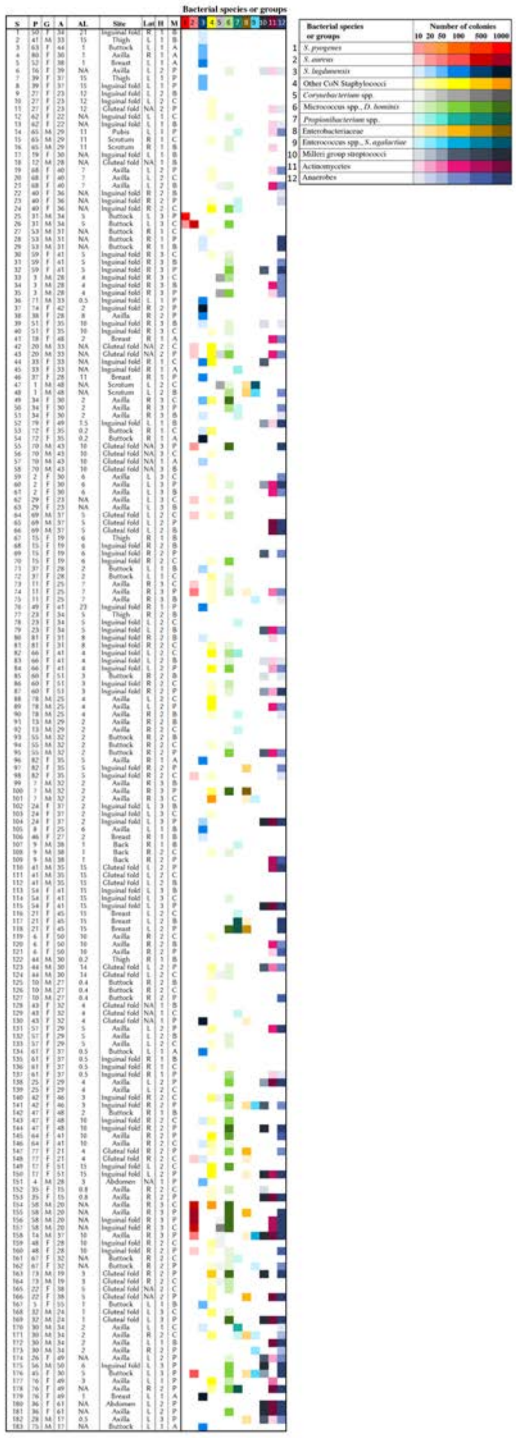

Technical Appendix Figure 1. Microbiological results of 183 samples collected from 82 patients in whom hidradenitis suppurativa was diagnosed. The 183 samples comprised 125 lesional samples (11 needle aspirations of abscesses, 49 biopsies of nodules or open suppurative lesions, and 65 swabs of open suppurative lesions) and 58 control samples performed by swabbing normal perilesional skin. S: Sample number; P: Patient number; G: Gender; A: Age (years); AL: Age of lesion (years); Lat: Laterality; H: Hurley stage of the lesion; M: Method of sampling (A, needle aspiration; B, biopsy; C, perilesional control swabs; P, purulent drainage swabs); NA, Data not available; CoN staphylococci : Coagulase negative staphylococci. Each line correspond to one sample. Each column correspond to one group of bacteria (indicated by a number and a color). The relative abundance of bacteria is indicated by a color gradient.

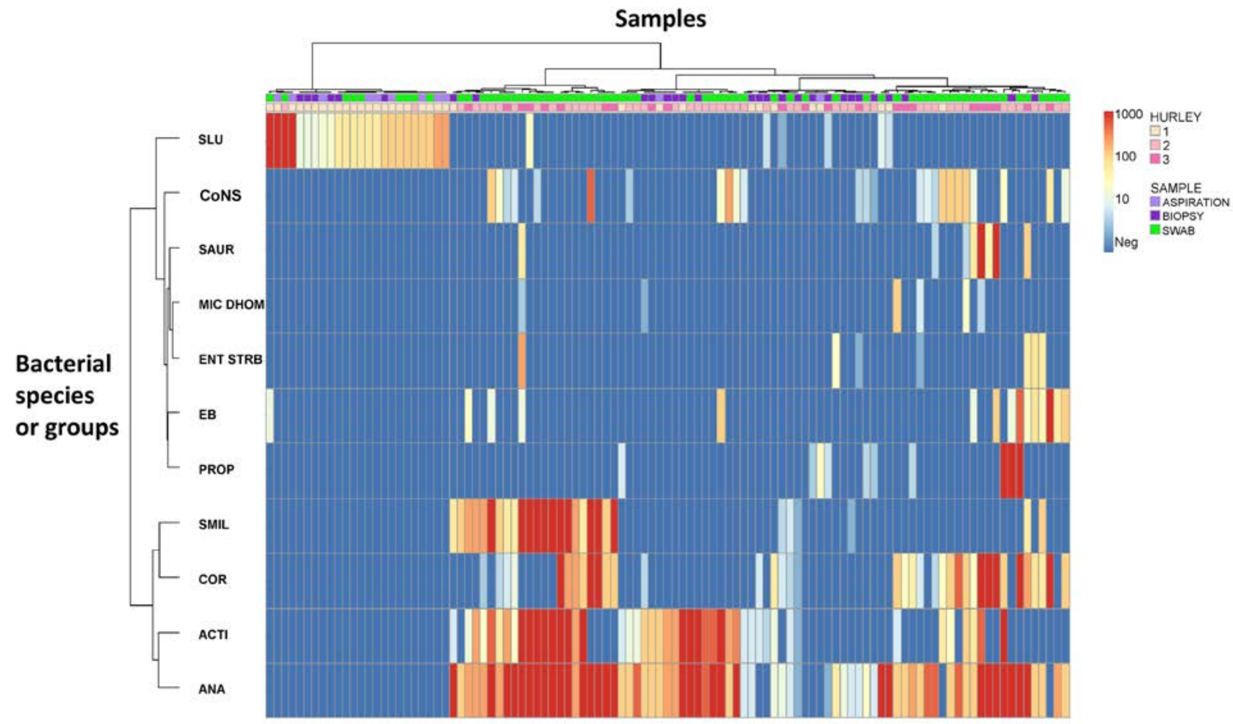

Technical Appendix Figure 2. Dendrogram of the bacterial flora associated with lesional samples. The bacterial isolates of 105 culture-positive lesional samples were used to perform hierarchical clustering (S. pyogenes lesional sample is not represented here). Each column represents a sample and each line represents a bacterial group. The nature of the sample (needle aspiration/biopsy/swab) is figured with a light purple or purple or green square at the top of the colored map. Hurley's staging is specified with a white/pink gradient for each sample. Each column represents a sample and each line represents a bacterial group. The relative abundance of a given bacterial group in a sample is indicated by a blue/red gradient SLU: Staphylococcus lugdunensis, CoNS: Coagulase negative staphylococci; SAUR: Staphylococcus aureus; MIC-DHOM: Micrococcus spp., Dermabacter hominis; ENT_STRB: Enterococci, Streptococcus agalactiae; PMIR_EB: Proteus mirabilis, enterobacteria; PROP: Propionibacterium spp.; SMIL: Streptococci of the Milleri group, COR: Corynebacterium spp.; ACTI: Actinomycetes, ANA: Strict anaerobes, STRA: Streptococcus pyogenes.

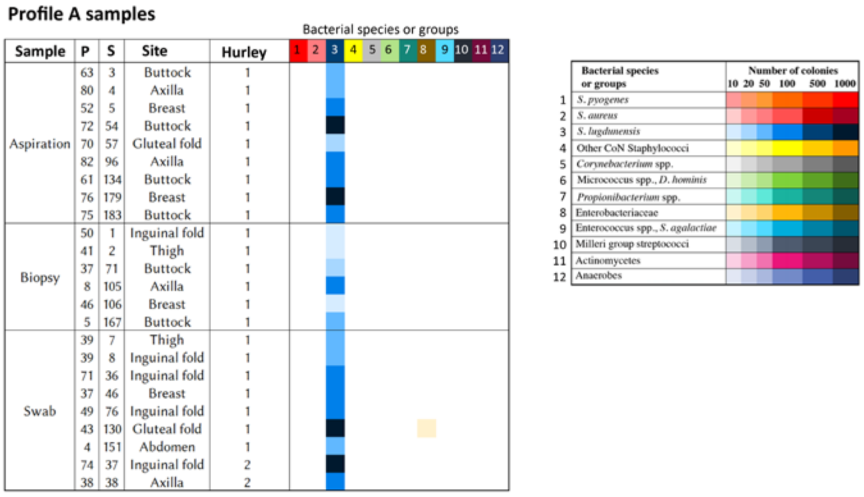

Profile A samples

Sample	P	S	Site	Hurley	1	2	3	4	5	6	7	8	9	10	11	12
Aspiration	63	3	Buttock	1												
	80	4	Axilla	1												
	52	5	Breast	1												
	72	54	Buttock	1												
	70	57	Gluteal fold	1												
	82	96	Axilla	1												
	61	134	Buttock	1												
	76	179	Breast	1												
	75	183	Buttock	1												
Biopsy	50	1	Inguinal fold	1												
	41	2	Thigh	1												
	37	71	Buttock	1												
	8	105	Axilla	1												
	46	106	Breast	1												
	5	167	Buttock	1												
Swab	39	7	Thigh	1												
	39	8	Inguinal fold	1												
	71	36	Inguinal fold	1												
	37	46	Breast	1												
	49	76	Inguinal fold	1												
	43	130	Gluteal fold	1												
	4	151	Abdomen	1												
	74	37	Inguinal fold	2												
	38	38	Axilla	2												

	Bacterial species or groups	Number of colonies 10 20 50 100 500 1000
1	*S. pyogenes*	
2	*S. aureus*	
3	*S. lugdunensis*	
4	Other CoN Staphylococci	
5	*Corynebacterium* spp.	
6	Micrococcus spp., *D. hominis*	
7	*Propionibacterium* spp.	
8	Enterobacteriaceae	
9	Enterococcus spp., *S. agalactiae*	
10	Milleri group streptococci	
11	Actinomycetes	
12	Anaerobes	

Technical Appendix Figure 3. Profile A: S. lugdunensis was the unique or predominant isolate in 24 of 106 positive cultures of 125 samples collected. P: Patient number; S: Sample number; CoN staphylococci: Coagulase negative staphylococci. Each line correspond to one sample. Each column correspond to one group of bacteria (indicated by a number and a color). The relative abundance of bacteria is indicated by a color gradient.

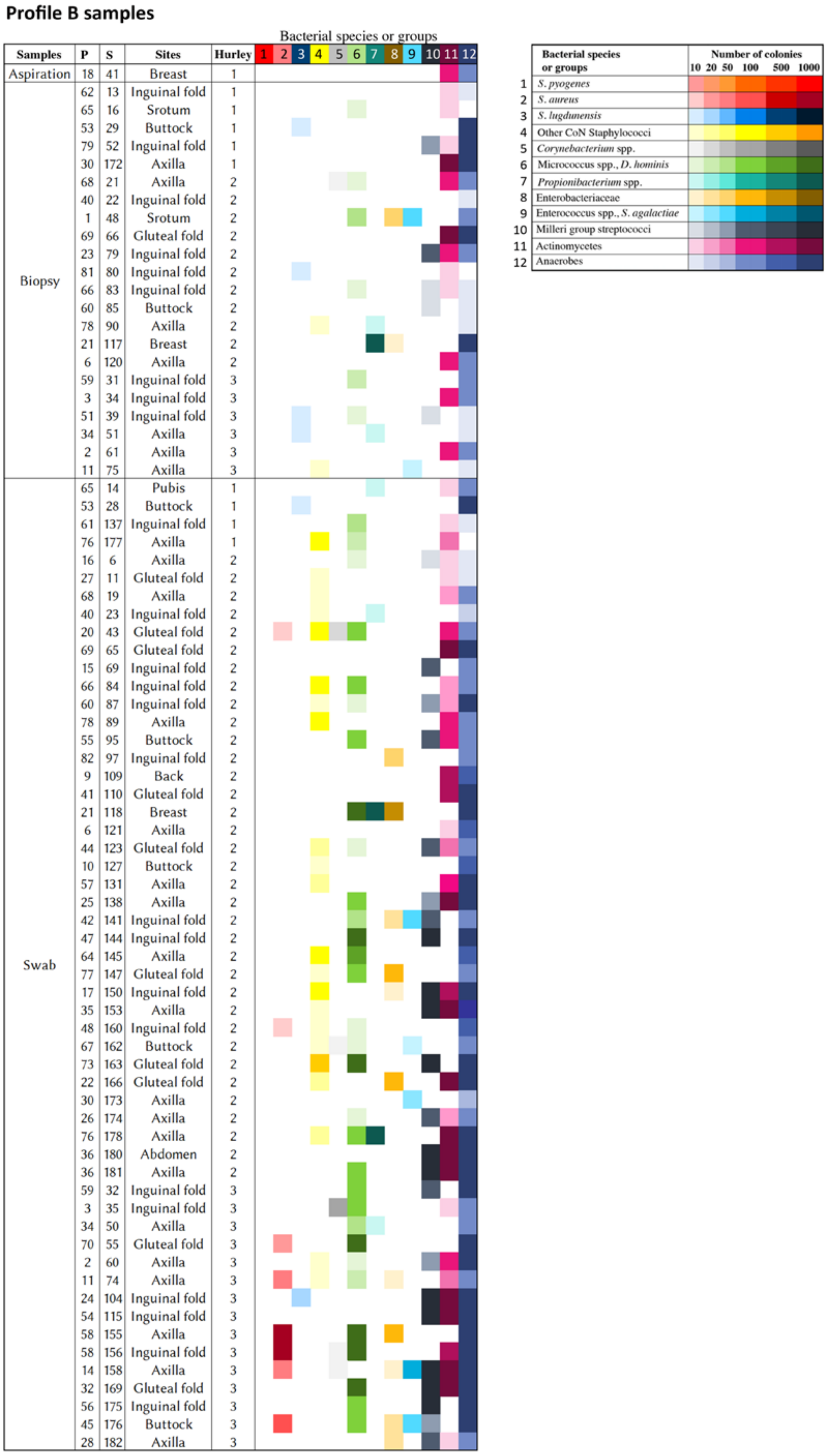

Technical Appendix Figure 4. Profile B: Mixed anaerobic flora composed by strict anaerobes and/ or actinomycetes and/or streptococci of the milleri group. This flora was inconstantly associated with coagulase negative staphylococci, corynebacteria, Propionibacterium spp., Dermabacter spp., micrococcaceae, enterobacteriaceae or Enterococcus spp. P: Patient number; S: Sample number; CoN staphylococci : Coagulase negative staphylococci. Each line correspond to one sample. Each column correspond to one group of bacteria (indicated by a number and a color). The relative abundance of bacteria is indicated by a color gradient.

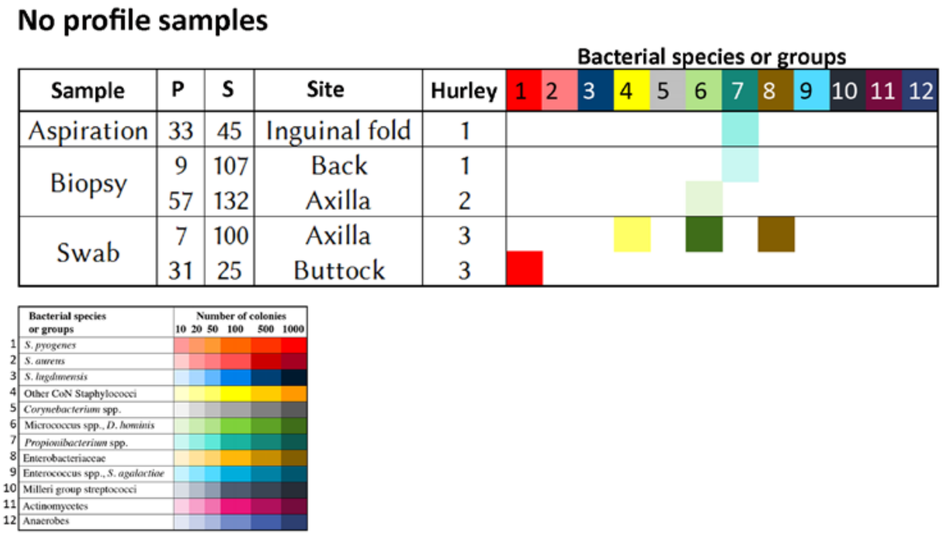

Technical Appendix Figure 5. No profile: samples corresponding neither to profile A nor to profile B. Each column corresponds to a bacterial species or group; each line corresponds to a lesion area. P: Patient number; S: Sample number; CoN staphylococci : Coagulase negative staphylococci. Each line correspond to one sample. Each column correspond to one group of bacteria (indicated by a number and a color). The relative abundance of bacteria is indicated by a color gradient.

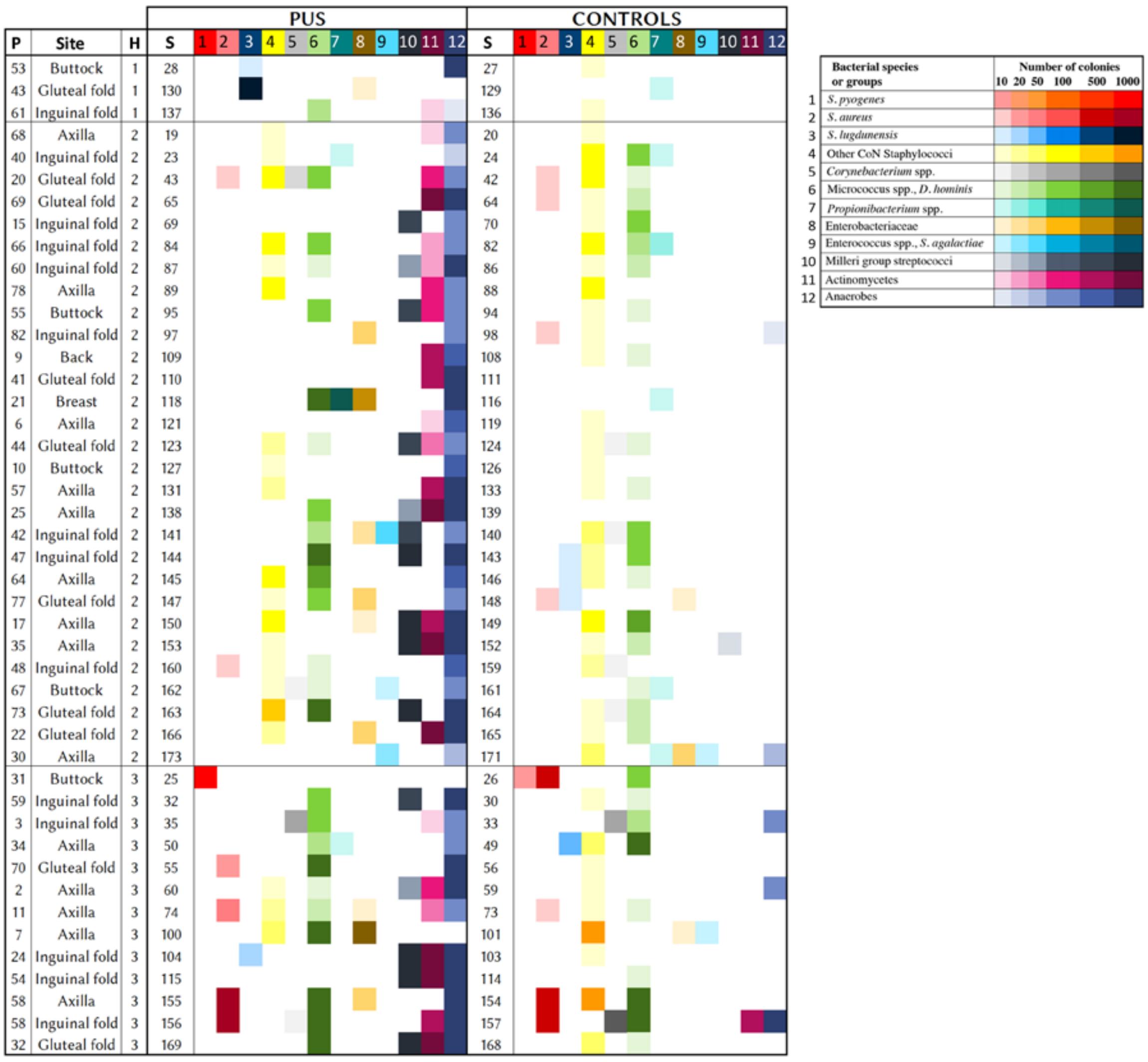

Technical Appendix Figure 6. Comparative culture results of 45 lesions swabs and corresponding normal skin perilesional controls. Each line corresponds to one lesion area. Lesions' and controls' culture results are presented on the left and right part of the table, respectively. Each column corresponds to one group of bacteria (indicated by a number and a color). The relative abundance of bacteria is indicated by a color gradient. P: Patient number; H: Hurley stage; S: Sample number; CoN staphylococci : Coagulase negative staphylococci.

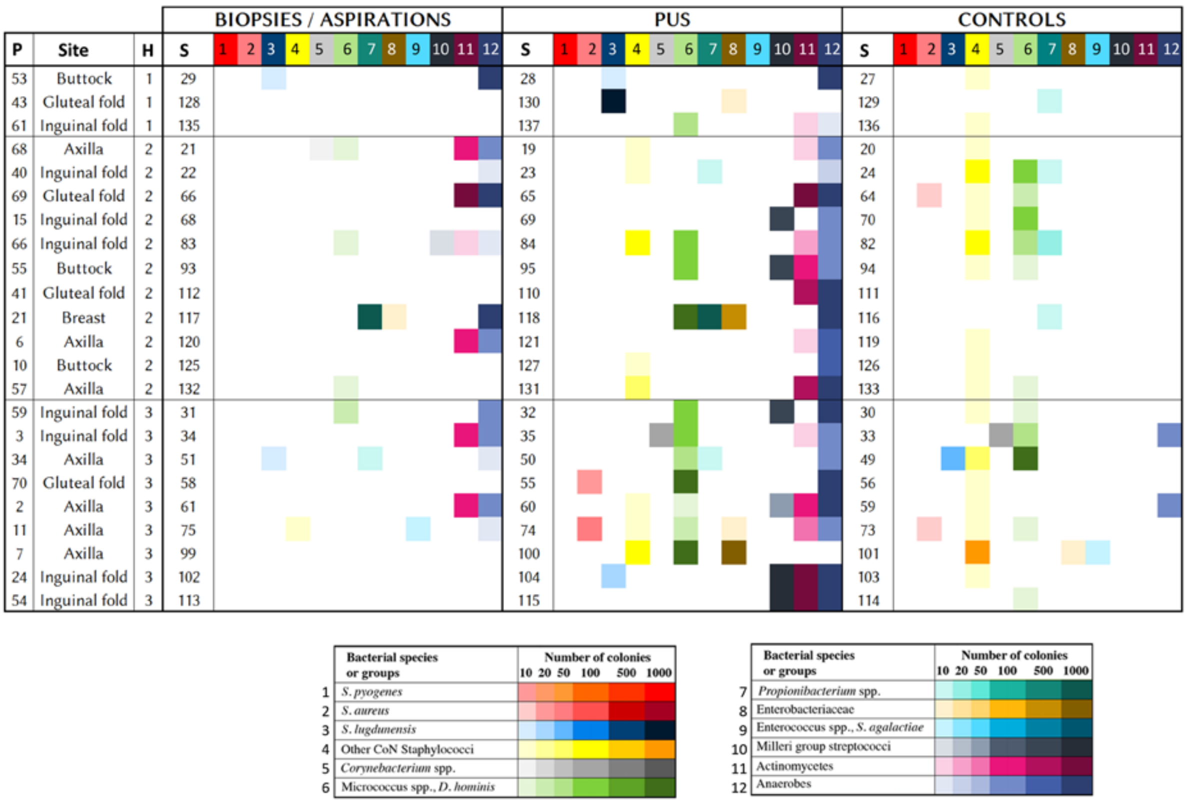

Technical Appendix Figure 7. Comparative culture results of biopsies/aspiration, purulent drainage, and perilesional control swabs of 23 open suppurating lesions. Each line corresponds to 1 lesion. Culture results of biopsies/ aspirations are presented on the left part of the table. Culture results of purulent drainage and perilesional swabs of the same lesions are presented on the middle and right part of the table. Each column correspond to one group of bacteria (indicated by a number and a color). The relative abundance of bacteria is indicated by a color gradient. P: Patient number; H: Hurley stage; S: Sample number; CoN: Coagulase negative. CoN staphylococci : Coagulase negative staphylococci.
